# Effect of Anticoagulants on Blood Cell Counts, Cell Morphology, and Leukocyte Immune Functions of Rainbow Trout (*Oncorhynchus mykiss*)

**DOI:** 10.3390/cells14171372

**Published:** 2025-09-02

**Authors:** Teresina De Iorio, Maria Carmela Scatà, Francesco Grandoni, Giovanna De Matteis, Arianna Martini, Nicolò Tonachella, Domitilla Pulcini, Fabrizio Capoccioni

**Affiliations:** Consiglio per la Ricerca in Agricoltura e l’Analisi Dell’Economia Agraria (CREA), Research Centre for Animal Production and Aquaculture, 00015 Monterotondo, RM, Italy; teresina.deiorio@crea.gov.it (T.D.I.); francesco.grandoni@crea.gov.it (F.G.); giovanna.dematteis@crea.gov.it (G.D.M.); arianna.martini@crea.gov.it (A.M.); nicolo.tonachella@crea.gov.it (N.T.); domitilla.pulcini@crea.gov.it (D.P.); fabrizio.capoccioni@crea.gov.it (F.C.)

**Keywords:** rainbow trout, cell viability, ROS, phagocytosis, flow cytometry, leukocytes, innate immunity

## Abstract

Hematological and immunological parameters may be influenced by various factors, including anticoagulants used to collect blood samples. This study aimed to evaluate the effect of three different anticoagulants (Li-Heparin, K_3_EDTA, and ACD-A) on rainbow trout blood cell count and morphology, leukocyte viability, ROS production, and phagocytosis. These evaluations were assessed through a combined approach based on flow cytometry and microscopy. Blood cell counts revealed differences in erythrocyte and thrombocyte count between anticoagulants (*p* < 0.001 and *p* < 0.01, respectively). Whole-blood samples collected in Li-Heparin displayed no hemolysis, in contrast to ACD-A and K_3_EDTA. A lower percentage of live cells and a higher percentage of apoptotic and dead cells were observed in K_3_EDTA compared to ACD-A (*p* < 0.05). Significantly lower ROS levels were observed in myeloid cells in Li-Heparin and ACD-A, compared to K_3_EDTA samples (*p* < 0.05). Overall, Li-Heparin better preserves rainbow trout blood cells in terms of hematological count, cell morphology, and ROS production and does not negatively affect the phagocytosis ability of leukocytes. Furthermore, the combined use of both flow cytometry and microscopy can provide a deeper insight into morpho-functional characteristics of leukocytes. Finally, we propose a flow cytometric assay to evaluate rainbow trout leukocyte viability, ROS production, and phagocytosis.

## 1. Introduction

Monitoring fish immune response is pivotal to maintain animal health and welfare and may contribute to improving aquaculture productivity. Blood contains easily accessible information about the individual physiological state of a fish [1]. The precise and constant assessment of blood parameters is an increasingly used approach to prevent the onset of diseases in farmed fishes. Thus, hematological parameters have become of great interest due to their ability to provide essential information to evaluate the immunological state of fish [2,3]. Blood tests represent a simple, comprehensive, and non-lethal approach to determine animal welfare, as blood is easy to collect and analyze. Nevertheless, these analyses currently require further investigation in fish since no universally accepted reference values have been established [4]. Indeed, blood cell counts in fish show seasonal variations and are regulated by environmental and life-stage parameters, such as water temperature, age, sex, and reproductive and nutritional status [4,5,6,7]. Moreover, the innate immune system parameters are key indicators of fish health and can be used to determine the effects of feed additives on fish welfare [8]. To date, phagocytosis efficiency has often been used as a central endpoint to evaluate innate immunity in fish [9], as well as in other vertebrates [10,11,12]. Indeed, it is important to obtain a reliable blood sample, which accurately represents the state of the animal, ensuring the quality of the data and the correct interpretation of results [13]. The ability to successfully prepare and preserve blood samples for microscopy and flow cytometry is critical to study the animal immune system [14]. The most important fish immunological responses to pathogens are phagocytosis and the related rapid production of ROS (oxidative burst), making them good indicators of cellular defense ability [15]. The use of an anticoagulant is a prerequisite for managing blood cells, due to the rapid formation of cellular clots. Therefore, the choice of an appropriate anticoagulant is a crucial requirement to achieve standard results [16,17]. The ideal anticoagulant has no effect on or minimally alters the characteristics and blood content of the organism. Some authors argue that the choice of anticoagulant for fish hematology is species-specific [18,19], since blood cells of various animals show different reactions to anticoagulants [20]. The effect of anticoagulants was previously observed on morphological, hematological, and biochemical parameters in snow trout (*Schizopyge plagiostomus*) [17], rainbow trout (*Oncorhynchus mykiss*) [19], and grey mullet (*Mugil cephalus*) [16]. The salts of ethylenediaminetetraacetic acid (EDTA) are widely used anticoagulants in routine hematology tests in humans, mammals, and other vertebrates, and have also shown some success in fish [6,19,21]. However, EDTA induces hemolysis in some species, causing deformation of the erythrocyte membrane and a decrease in the viability of fish leukocytes [21,22,23]. In contrast, heparin is mainly employed in clinical biochemistry in mammals, since it has minimal chelating properties, minimal interference with water, and a relatively low cation concentration. Three heparin salts are most commonly used for blood sample collection: sodium heparin, ammonium heparin, and lithium heparin. Several studies suggested the use of lithium heparin for hemocytometry of animals with nucleated erythrocytes, due to the absence of hemolysis in samples treated with this salt. Indeed, many authors described heparin as the anticoagulant of choice in fish hematology [19,22,24,25]. On the other hand, Acid Citrate Dextrose (ACD-A) is the only anticoagulant to date approved by the Food and Drugs Administration (FDA) in blood banks to preserve blood for transfusion in humans and mammals, due to its ability to promote cell survival for up to 42 days [26]. ACD and EDTA act similarly by chelating calcium in the blood, inhibiting clot formation, although EDTA binds calcium more strongly and irreversibly [27]. Since calcium is pivotal for cell-to-cell interactions, Heparin and ACD anticoagulants were demonstrated to be more effective than EDTA in leukocyte functional studies [27].

To the best of our knowledge, no information is available regarding ACD-A’s effect on blood cells in fish. Moreover, no guidelines are published on the most appropriate anticoagulant used in functional studies on fish leukocytes, and no comparative evaluation of the effects of different anticoagulants on leukocyte viability, reactive oxygen species (ROS) detection, and phagocytosis in rainbow trout has been carried out. To fill this gap, this study aimed to evaluate and compare the effects of three commonly used anticoagulants (ACD-A, K_3_EDTA, and Li-Heparin) on hematological parameters (blood cell count and cell morphology) and immunological functions (leukocyte viability, ROS production, and phagocytosis ability) of rainbow trout cells. These parameters were analyzed through a combined approach, based on flow cytometry and microscopy, integrating both techniques to assess the influence of anticoagulants on blood cell morpho-functional characteristics.

## 2. Materials and Methods

### 2.1. Ethical Statement

This study complies with the European Union (EU) Directive 2010/63/EU on the protection of animals used for scientific purposes. All the procedures included in this study were performed in line with national laws and institutional guidelines and were approved by the committee responsible for animal welfare at the CREA Animal Production and Aquaculture Research Centre (Authorization number: 00017431; 29 February 2024).

### 2.2. Rainbow Trout Farming Conditions and Peripheral Blood Sampling

For this study, rainbow trout (*O. mykiss*) from a common batch weighing between 400 and 700 g were reared in a commercial rectangular pond (Rieti, Central Italy), at an average water temperature of 16 ± 0.2 °C and a pH of 8 ± 0.2. Trout were fed twice a day with a commercial pellet diet (Skretting, Stavanger, Norway, Optiline 3P). Six rainbow trout were selected according to Vallejos-Vidal [28], caught randomly using a scoop net, and immediately euthanized by the farmer, according to national laws regarding commercial fish slaughtering. Subsequently, 10 mL of peripheral blood from each fish was collected from the caudal vein using a syringe with a 21G needle (Beckton Dickinson, Franklin Lakes, NJ, USA) and immediately aliquoted in commercially available vacutainer tubes containing the three selected anticoagulants: spray-dried Li-heparin, K_3_EDTA, or liquid ACD-A (Beckton Dickinson). The tubes were labeled, placed in an expanded polystyrene box with a gel pack, and transported to the laboratory following a carefully maintained cold chain.

### 2.3. Evaluation of Blood Cell Count and Cell Morphology

Blood cell count was manually established by a Neubauer hemocytometer (MUHWA Scientific, Shanghai, China) on whole blood diluted 1:200 in Natt–Herrick’s stain solution, as previously described by Nabi [4]. Cell morphology was then assessed on Hemofast-stained blood smears. Briefly, slides were prepared by dispensing whole blood diluted 1:2 in Dulbecco’s phosphate-buffered saline (DPBS; BioConcept, Allschwil, Switzerland) or a suspension of peripheral blood leukocytes (PBLs) (1 × 10^6^ cells/mL; see Section 2.4). The samples were air-dried and fixed with 4% formaldehyde solution in DPBS for 10 min. Slides were then washed in deionized water (diH_2_O) and stained for 30 s in Wright’s modified solution containing Eosin Y and methylene blue (Hemofast, Mascia Brunelli, Milan, Italy). Samples were then rinsed in diH_2_O for 1 min, mounted with Mount Quick (BioOptica, Milan, Italy), and air-dried. Cell morphology was analyzed at 400× magnification under an Axioplan light microscope (Zeiss, Oberkochen, Germany).

### 2.4. Evaluation of Viability in Rainbow Trout Peripheral Blood Leukocytes (PBLs)

PBLs were prepared by hypotonic lysis of erythrocytes as previously described [29]. This protocol was validated for heparinized blood, and since we observed consistent results with EDTA-treated samples in both flow cytometry and microscopy, we maintained the same lysis conditions for both anticoagulants. In the present manuscript, we used the ACD-A anticoagulant for the first time in rainbow trout; therefore, we previously optimized lysis conditions to balance efficient RBC removal with the preservation of leukocyte integrity, despite its use has been limited to microscopy analysis. Briefly, 2 mL of refrigerated whole blood was dispensed in a 50 mL tube containing 18 mL of cold cell culture-grade water and lysed by inversion for 20 s. Then, 2 mL of 10× DPBS was added to restore isotonicity. The tube was kept on ice for 10 min, after which the PBLs were easily separated from nuclear clump material by passing through a 100 µm cell strainer (Sarstedt, Nümbrecht, Germany). PBLs were pelleted by centrifugation (300× *g* for 5 min at 4 °C) and washed once with Leibovitz’s medium (L15, Gibco, Waltham, MA, USA) supplemented with 1% Fetal Calf Serum (FCS, Gibco). The PBLs were resuspended in L15 medium supplemented with 10% FCS, counted by the LUNA-II™ Automated Cell Counter (Logos Biosystems, Anyang, South Korea), and adjusted to 1 × 10^7^ cells/mL. The Dead Cell Apoptosis Kit with Annexin V FITC & Propidium Iodide (Thermo Fisher Scientific, Waltham, MA, USA) was used to measure the apoptosis and viability of cells, according to the manufacturer’s instructions. Labeled samples were immediately collected on a CytoFLEX flow cytometer (Beckman Coulter, Brea, CA, USA). Data were analyzed with Kaluza Analysis software v 2.1 (Beckman Coulter). For fluorescence microscopic analyses, stained cells were deposited onto a glass slide and mounted with ProLong™ Gold antifade mounting media (Thermo Fisher Scientific, Waltham, MA, USA). The samples were analyzed at 400× magnification under Aristoplan (Leitz, Wetzlar, Germany) equipped with 450/490 and 510/630 BP, and a CMOS digital camera (Hayear, Shenzhen, China). Cells that showed no Annexin V staining of the cellular membrane were considered alive, whereas apoptotic cells displayed a high degree of membrane labeling. Cells showing strong PI nuclear staining were considered dead.

### 2.5. Evaluation of Phagocytosis and Reactive Oxygen Species (ROS) Production in Rainbow Trout Peripheral Blood Leukocytes

**Figure 1:**
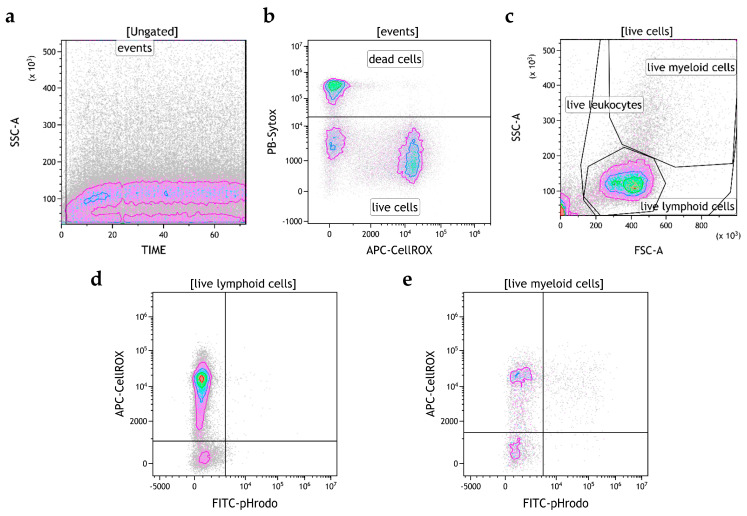
Gating strategy used to evaluate the production of ROS in live phagocytic cells. Events were gated in the dot plot time vs. SSC-A to exclude bursts (**a**); dead cells were excluded with the dot plot PB-Sytox vs. APC-CellROX (**b**). Live cells were then gated for lymphoid and myeloid cells based on FSC and SSC properties (**c**). The dot plot FITC-pHrodo vs. APC-CellROX was used to identify the ROS^+^/pHR^+^ lymphoid (**d**) and myeloid (**e**) populations (in upper-right quadrant). These dot plots are representative of one sample.

The pHrodo^TM^ Green Conjugated *E. coli* Bioparticles^®^ and CellROX^®^ Deep Red Assay Kit (Thermo Fisher Scientific) were used to evaluate phagocytosis activity and ROS production in leukocytes (Section 2.4). These two assays have been tested both as single assays and as a combined assay for rapid and simultaneous detection. The pHrodo™ Green Conjugated *E. coli* Bioparticles^®^ were opsonized with 30% autologous plasma in PBS for 60 min at room temperature and centrifuged at 800× *g* for 10 min at 4 °C. PBLs (5 × 10^5^/50 μL) were incubated with a 20:1 particle/cell ratio of opsonized pHrodo ™ Green *E. coli* for 2 h at 16 °C, washed once with PBS 1% BSA, and pelleted by centrifugation (300× *g* for 5 min at 4 °C). These cells were collected on a CytoFLEX flow cytometer (Beckman Coulter). Afterward, to perform the simultaneous assay, PBLs (5 × 10^5^/500 μL) were incubated with 500 nM of CellROX^®^ reagent for 30 min at RT. During the final 15 min, 1 µM of SYTOX^®^ Blue Dead Cell reagent was added. Samples were immediately collected on a flow cytometer using a 488 nm excitation for the pHrodo™ Green *E. coli* Bioparticles^®^, a 405 nm excitation for the SYTOX^®^ Blue Dead Cell reagent, and a 638 nm excitation for CellROX^®^ Deep Red reagent. A single assay without pHrodo™ Green Conjugated *E. coli* Bioparticles^®^ was performed to evaluate basal ROS levels. Fluorescence emission was collected using 525/40, 450/45, and 660/20 BP filters, respectively. At least 100,000 leukocytes were measured after gating according to the forward/side-scattered (FCS/SSC) characteristics. Kaluza Analysis software v 2.1 (Beckman Coulter) was used to analyze the flow cytometric data. Dot plots showing FSC vs. SSC were set up to differentiate lymphoid and myeloid cells by size, according to the gating strategy depicted in Figure 1.

For the fluorescence microscopy analysis, stained cells were deposited onto a glass slide, air-dried, fixed in 4% formaldehyde for 10 min, and mounted with ProLong™ Gold antifade mounting medium with DAPI. Samples were observed at 400× magnification, under an Axioplan microscope (Zeiss) equipped with 395/440, 450/490, and 620/670 BP filters and a CMOS digital camera (Hayer, Shenzhen, China). Images were taken with constant exposure parameters. A total of 15 randomly selected fields at 400× total magnification were analyzed for each specimen. Fluorescence intensity was evaluated by measuring particle intensity with ImageJ analysis software v 1.54 (https://imagej.net/ij/, accessed on 27 August 2025)) and is expressed as relative fluorescence units (RFU). The PBL ROS production and phagocytosis were estimated by counting the total number of cells and the positive cells, through the ImageJ manual cell counter plugin, and are expressed as percentages. Lymphoid and myeloid cells were defined according to their dimension and nuclear morphology: small cells with rounded nuclei were considered lymphoid; large cells with either indented, half-moon, or multilobed nuclei were defined as myeloid. Since the blue-violet filter covers the wavelength of both DAPI and SYTOX^®^, the ROS production was evaluated on total PBLs.

### 2.6. Statistical Analysis

The fluorescence intensity and the percentage of positive cells, obtained by microscopy and by flow cytometry, are presented as boxplot diagrams, where the box represents the interquartile range (IQR), the line is the median, the central point characterizes the mean, and the box edges are the 25th and 75th quartiles. Experimental groups’ means were compared using the nonparametric Kruskal–Wallis test, with Bonferroni correction. Percentages of cell counts were analyzed using the Chi-square test, with a Bonferroni-corrected *p*-value. Differences of *p* ≤ 0.05 were considered significant and are indicated with different superscripts. The adjusted threshold was set at α = 0.05/3 (0.017) when Kruskal–Wallis’ test was applied, while α = 0.05/9 (0.006) was used when Chi-square was exploited, to reduce the false positive rate while improving the likelihood of results being reproducible. All the statistical tests were performed using the freely available software Past v. 4.17 (see https://www.nhm.uio.no/english/research/resources/past/, (accessed on 7 July 2024)) [28].

## 3. Results

### 3.1. Anticoagulants’ Effects on Blood Cell Counts and Cell Morphology

A significant decrease in RBC count was shown in ACD-A and K_3_EDTA compared to the Li-Heparin anticoagulant. Moreover, a significant decrease in thrombocyte (TBC) count was observed in ACD-A compared to Li-Heparin and K_3_EDTA. No significant differences were found in other cellular parameters (Table 1).

**Figure 2:**
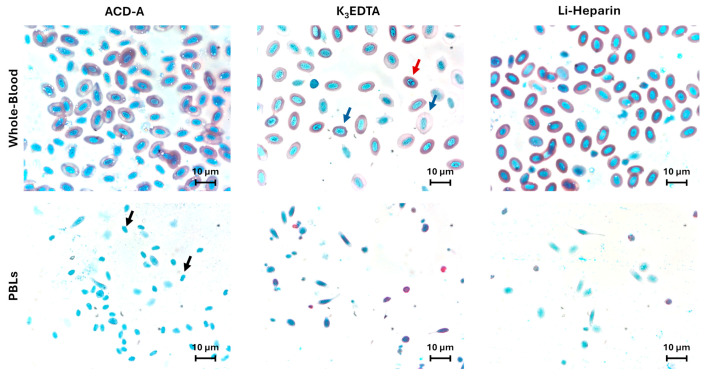
Representative cell morphology images of Hemofast-stained smear from samples collected in ACD-A, K_3_EDTA, and Li-Heparin. The top panel displays whole-blood cells, while the bottom panel shows isolated PBL morphology. The black arrows show naked erythrocyte nuclei in ACD-A samples, and the red and blue arrows indicate, respectively, anisonucleosis and anisocytosis. Scale bar: 10 µm.

Our results showed erythrocyte hemolysis in whole-blood specimens collected in ACD-A and K_3_EDTA, while Li-Heparin displayed no erythrocyte hemolysis (Figure 2).

Moreover, the K_3_EDTA group displayed anisocytosis (unequal cell size) and anisonucleosis (unequal nucleus size), while Li-Heparin showed a negligible effect on cell morphology. PBLs prepared from ACD-A samples displayed naked nuclei, released by a non-optimal lysis of erythrocytes, which were absent in K_3_EDTA and Li-Heparin (Figure 2).

### 3.2. Anticoagulants’ Effects on Leukocyte Viability

**Figure 3:**
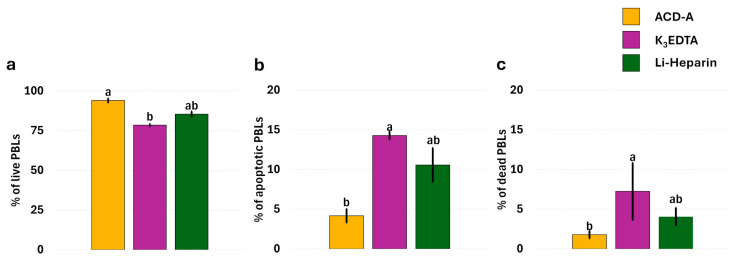
Effect of anticoagulants on PBL viability of rainbow trout. Microscopic analysis of live (**a**), apoptotic (**b**), and dead (**c**) leukocytes. Data are presented as means; error bars represent the standard deviation. Treatments with different superscript letters demonstrate significant differences (Chi-square test; *p* < 0.05; α = 0.006).

All the functional evaluations were performed through a combined approach: flow cytometry and fluorescence microscopy. Since no optimal lysis of erythrocytes was detected in ACD-A samples, we decided not to analyze the flow cytometric data of these samples. The flow cytometric analysis showed no significant differences (*p* = 0.5218) in leukocyte viability between Li-Heparin and K_3_EDTA samples, according to fluorescence microscopy data. The microscopic evaluation of cell viability revealed a greater percentage of live PBLs (Figure 3a) and a lower percentage of apoptotic (Figure 3b) and dead cells (Figure 3c) in ACD-A compared to K_3_EDTA samples. Samples collected in Li-Heparin displayed intermediate values.

### 3.3. Anticoagulant Effects’ on Basal ROS Levels in PBLs: Single Assay

**Figure 4:**
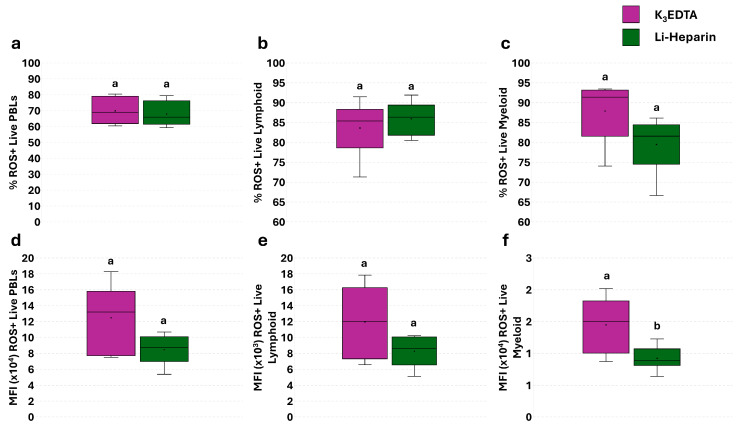
Flow cytometric analysis of ROS basal levels in PBLs from K_3_EDTA and Li-Heparin detected by single assay. Percentage (**a**–**c**) and MFI (**d**–**f**) of live ROS^+^ PBLs, lymphoid cells, and myeloid cells. Treatments with different superscript letters show significant differences (percentage of positive cells was analyzed with Chi-square test; *p* < 0.05; α = 0.006; MFI was analyzed with Kruskal–Wallis’ test; *p* < 0.05; α = 0.017).

A single assay without pHrodo™ was performed in all samples to assess the basal level of ROS. No significant differences were detected by flow cytometry in the percentage of total ROS^+^ live PBLs, and lymphoid and myeloid subpopulations (Figure 4a–c). A significant increase in the mean fluorescence intensity (MFI) of ROS^+^ myeloid cells was observed in K_3_EDTA compared to Li-Heparin samples (Figure 4f).

**Figure 5:**
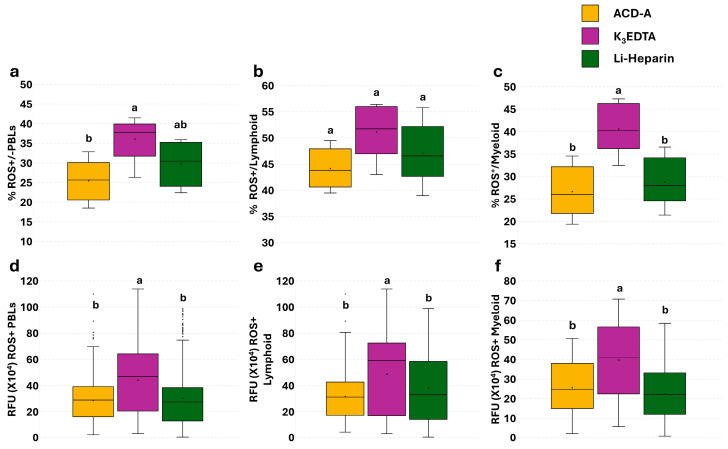
Microscopic analysis of ROS basal levels in PBLs from K_3_EDTA and Li-Heparin detected by single assay. Percentage (**a**–**c**) and RFU value (**d**–**f**) of ROS^+^ total PBLs, lymphoid cells, and myeloid cells. Treatments with different superscript letters display significant differences (percentage of positive cells was analyzed with Chi-square test; *p* < 0.05; α = 0.006; MFI was analyzed with Kruskal–Wallis test; *p* < 0.05; α = 0.017).

Since the microscopic definition of lymphoid and myeloid cell populations was also based on their nuclear morphology, the ROS levels were assessed on total cells, without dead cell exclusion. Total PBLs and myeloid subpopulations isolated from K_3_EDTA samples showed a higher percentage (Figure 5a,c) and RFU value (Figure 5d,f) of ROS^+^ cells, compared to Li-Heparin- and ACD-A-treated samples. The RFU ROS^+^ value was significantly higher in K_3_EDTA, compared to Li-Heparin and ACD-A (Figure 5e).

### 3.4. Effect of Anticoagulants on Phagocytosis in PBLs: Single Assay

**Figure 6:**
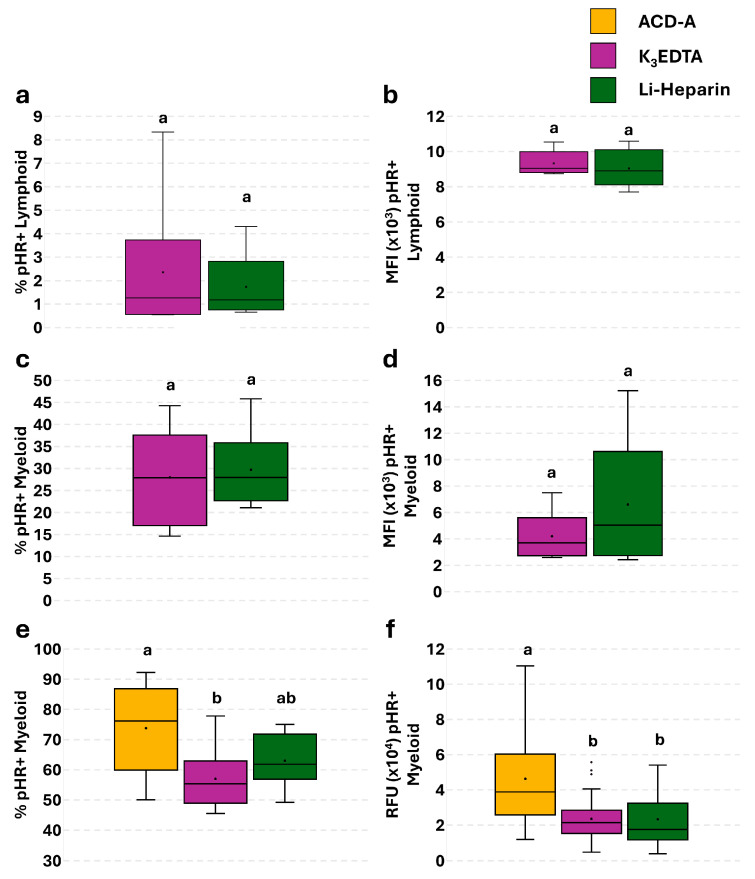
Flow cytometry and fluorescence microscopy analyses of anticoagulants’ effect on rainbow trout PBL phagocytosis ability. Percentage (**a**,**c**) and MFI (**b**,**d**) of pHR^+^ lymphoid and myeloid cells assessed by flow cytometry. Percentage (**e**) and RFU value (**f**) of pHR^+^ myeloid cells assessed by fluorescence microscopy. Treatments with different superscript letters demonstrate significant differences (percentage of positive cells was analyzed with Chi-square test; *p* < 0.05; α = 0.006; MFI and RFU were analyzed with Kruskal–Wallis test; *p* < 0.05; α = 0.017).

To evaluate the anticoagulants’ effect on phagocytosis ability, a single assay using the pHrodo^TM^ Green *E. coli* Bioparticles^®^ kit was performed. Flow cytometric evaluation of phagocytic cells (Figure 6a–d) showed no significant differences in the percentage (Figure 6a,c) or in the MFI (Figure 6b,d) of pHR^+^ lymphoid (Figure 6a,b) and myeloid (Figure 6c,d) subpopulations. These data were confirmed by microscopic analyses, displaying a greater percentage of pHR^+^ myeloid cells in ACD-A compared to K_3_EDTA (Figure 6e), and a higher RFU value compared to Li-Heparin and K_3_EDTA (Figure 6f).

No positive cells were detected in lymphoid subpopulations by fluorescence microscopy.

### 3.5. Effect of Anticoagulants on Phagocytosis and Phagocytosis-Related ROS Production in PBLs: The Flow Cytometry and Fluorescence Microscopy Combined Approach

**Figure 7:**
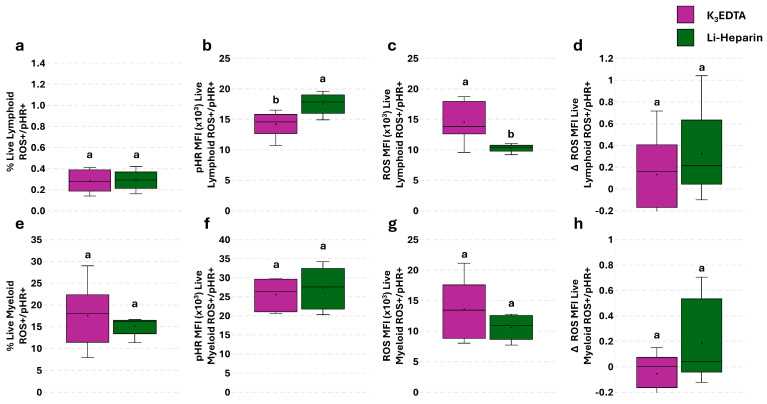
Flow cytometry analysis of anticoagulants’ effect on rainbow trout PBL phagocytosis and related oxidative stress. Percentage of ROS^+^/pHR^+^ live lymphoid cells (**a**). pHR-associated MFI of ROS^+^/pHR^+^ (**b**), ROS-related MFI of ROS^+^/pHR^+^ live lymphoid cells (**c**), and phagocytosis-related ROS MFI (Δ) signal normalized through basal ROS values in live lymphoid cells (**d**). Percentage of ROS^+^/pHR^+^ live myeloid cells (**e**). pHR-associated MFI of ROS^+^/pHR^+^ live myeloid cells (**f**), ROS-related MFI of ROS^+^/pHR^+^ live myeloid cells (**g**), and ROS-related MFI (Δ) signal normalized through basal ROS values in live myeloid cells (**h**), determined by the single assay without pHR *E. coli* Bioparticles. Treatments with different superscript letters show significant differences (percentage of positive cells was analyzed with Chi-square test; *p* < 0.05; α = 0.006; MFI was analyzed with Kruskal–Wallis test; *p* < 0.05; α = 0.017).

A simultaneous assay to determine cellular innate immune function was assessed to improve the informativeness and power of the single phagocytosis and ROS production assays. The flow cytometric analysis revealed no differences in the percentage of live lymphoid and myeloid ROS^+^/pHR^+^ cells (Figure 7a,e). A significant increase was detected in pHR MFI of live lymphoid ROS^+^/pHR^+^ cells in Li-Heparin compared to K_3_EDTA (Figure 7b). The opposite trend was detected in ROS MFI of live lymphoid ROS^+^/pHR^+^ cells (Figure 7c). No significant differences were observed in ROS MFI of myeloid cells (Figure 7f,g).

**Figure 8:**
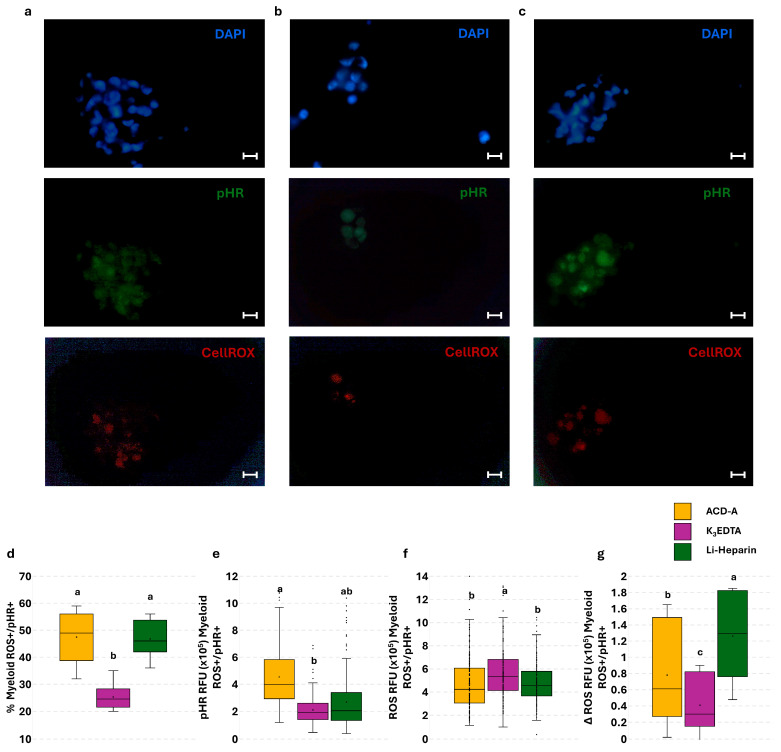
Fluorescence microscopy evaluation of anticoagulants’ effect on rainbow trout PBL phagocytosis and related oxidative burst. Representative fluorescence microscopy images show cell nuclei (blue, top panel), engulfed bacteria (green, central panel), and ROS-producing cells (red, bottom panel) in ACD-A (**a**), K_3_EDTA (**b**), and Li-Heparin (**c**) (Scalebar: 10 µm). Percentage of ROS^+^/pHR^+^ (**d**), pHR RFU of ROS^+^/pHR^+^ (**e**), and pHR RFU of ROS^+^/pHR^+^ (**f**) in myeloid cells. ROS-related RFU signal (Δ) after normalization with basal ROS values determined in myeloid cells by the single assay without pHR *E. coli* Bioparticles (**g**). Treatments with different superscript letters display significant differences (percentage of positive cells was analyzed with Chi-square test; *p* < 0.05; α = 0.006; RFU was analyzed with Kruskal–Wallis test; *p* < 0.05; α = 0.017).

To highlight phagocytosis-associated ROS production, we calculated the increase in ROS amount (Δ) after the stimulation with bacteria compared to the basal levels. The Δ ROS MFI of live lymphoid and myeloid ROS^+^/pHR^+^ cells revealed no significant differences, although Li-Heparin showed an increased trend in ROS production, compared to K_3_EDTA (Figure 7d,h), according to microscopic evaluation (Figure 8g). Microscopic examination of all leukocytes suggested higher phagocytosis ability and efficiency in specimens treated with ACD-A (Figure 8a) and Li-Heparin (Figure 8c), compared to those collected in K_3_EDTA (Figure 8b). The K_3_EDTA-preserved samples showed a lower degree of PBL aggregation (leucoagglutination; Figure 8b), compared to other tested anticoagulants (Figure 8a,c). These observations were confirmed by the higher percentage and the greater pHR RFU value of myeloid ROS^+^/pHR^+^ cells (Figure 8d,e) detected in ACD-A and Li-Heparin samples compared to K_3_EDTA. On the contrary, myeloid ROS^+^/pHR^+^ cells displayed a higher ROS RFU value in K_3_EDTA (Figure 8f). Li-Heparin samples showed a greater Δ ROS RFU value of myeloid ROS^+^/pHR^+^ than the other anticoagulants (Figure 8g).

## 4. Discussion

In the present study, the effects of ACD-A, K_3_EDTA, and Li-Heparin on rainbow trout hematological parameters (blood cell count and cell morphology) and immunological functions (leukocyte viability, ROS production, and phagocytosis ability) were evaluated and compared. Blood cell counts and cell morphology were influenced by anticoagulants. More specifically, Li-Heparin samples displayed no hemolysis, while ACD-A and K_3_EDTA affected RBC count due to erythrocyte hemolysis, in agreement with Sheikh and Ahmed [17]. Furthermore, EDTA samples displayed morphological alterations in erythrocytes, mainly anisocytosis and anisonucleosis. Similar results were observed in previously published studies, describing a sharp decrease in RBC counts, caused by hemolysis, paralleled by erythrocyte swelling, anisocytosis, and anisonucleosis in EDTA-treated samples of rainbow trout [19] and common carp Cyprinus carpio [20,23]. Moreover, the ACD-A samples decreased the TBC count and increased the proportion of thrombocytes with fusiform morphology. Although ACD-A had never been used in fish hematology, in human blood samples, this anticoagulant causes a decrease in platelet count in comparison to EDTA-stored samples [30]. This effect is probably due to the citrate’s ability to induce ‘platelet micro-aggregates’ [31,32]. These observations could explain the decrease in TBC value detected in ACD samples. According to Walencik and Witeska [23], our results displayed no significant variation in either white blood cell (WBC) counts or their subpopulations’ counts or leukocyte morphology between anticoagulants.

Regarding anticoagulants’ effects on immunological functions, the ACD-A samples displayed higher PBL viability compared to K_3_EDTA, associated with lower apoptosis. To the best of our knowledge, no evidence is available regarding ACD-A’s influence on rainbow trout leukocyte viability. However, our data agree with Carter and colleagues [33], who demonstrated enhanced leucocyte viability in mammalian blood collected in ACD and heparin compared to EDTA samples. Other authors showed that EDTA significantly affects rainbow trout leukocyte proliferation ability [34] and leukocyte viability of other teleosteans, among them being the shanny Lipophrys pholis (formerly *Blennius pholis*) [22] and the South American catfish *Rhamdia quelen* [35]. A possible explanation of this effect could reside in the EDTA-mediated increase in blood pH values [25] or in the Ca^2+^ chelation, which causes an altered permeability of cell membranes [20], which could affect cell viability.

Cellular ROS production is often used to evaluate cell oxidative stress in mammals as well as in fish species [23,36,37,38]. Interestingly, ROS production was influenced by anticoagulants. More specifically, flow cytometric data highlighted that live PBLs derived from EDTA samples showed higher ROS levels compared to Li-Heparin, although significant differences were found only in the myeloid cell population. Furthermore, fluorescence microscopy revealed higher ROS content in myeloid cells in EDTA samples. This result fits well with Walenik and Witeska [23], who showed increased ROS levels in common carp blood samples collected in EDTA tubes, compared to those in heparin and citrate. This effect may be explained by EDTA-induced hemolysis, which promotes phagocytosis of RBC debris [23,25,37].

The analysis of phagocytosis is usually conducted using fluorochrome-labeled bacteria or bioparticles. Recently, the pHrodo^TM^ *E. coli* Bioparticles^®^ conjugates were validated to assess phagocytosis [12]. These bioparticles are fluorescent in the phagolysosome acidic microenvironment, thus eliminating the need for washing and quenching steps [10]. This assay has already been validated by imaging flow cytometry of fish blood and shellfish hemolymph, providing promising and reliable results [39]. Our flow cytometric data revealed no statistical differences in myeloid and lymphoid cell phagocytosis between Li-Heparin and K_3_EDTA samples. Since an incomplete lysis of erythrocytes occurred in ACD-A samples, the flow cytometry data were not included in this study. However, ACD-A’s effect on PBL phagocytosis was evaluated by fluorescence microscopy, due to the clearly defined erythrocyte nuclear morphology. A significantly higher percentage and RFU value of pHR+ myeloid cells were observed in ACD-A compared to K_3_EDTA. Flow cytometry allows us to increase the ability to explore immune cell functions via a multiparametric approach that, at the same time, evaluates viability, ROS production, and phagocytosis only in live cells. The effect of different anticoagulants was also evaluated, for the first time, in this simultaneous assay. Flow cytometric data highlighted significantly increased values of pHR MFI in ROS^+^/pHR^+^ live lymphoid cells in Li-Heparin compared to K_3_EDTA samples, and an opposite trend was detected in ROS MFI in the same cell subpopulation. The same trend was observed in the myeloid cell population, although no significant differences were noted. In agreement with this, the microscopic analysis revealed greater myeloid cell phagocytosis activity in ACD-A and Li-Heparin, compared to K_3_EDTA. Furthermore, myeloid cells collected in Li-Heparin showed an increased phagocytosis-related ROS production (Δ ROS RFU), suggesting an enhanced ability of these cells to destroy bacteria. Notably, we also detected myeloid cell aggregation (leucoagglutination) in all the anticoagulants tested after incubation with pHrodo *E. coli* Bioparticles, although this cell behavior is observed less in K_3_EDTA (5-fold lower). Human studies have already described leucoagglutination associated with autoimmune disease, immunosuppression, and infection/sepsis [40,41,42,43]. In our study, this phenomenon is observed less in K_3_EDTA, suggesting less efficient cell-to-cell communication in these samples. Consistently with this, EDTA has already been described to inhibit phagocytosis in rainbow trout leukocytes, although at approximately 10-fold-higher concentration (0.5 M), than the K_3_EDTA vacutainer [44]. Nevertheless, due to the chelating activity of K_3_EDTA, we could speculate that the absence of calcium in the microenvironment, which is a co-factor of adhesion proteins, may not only prevent leucoagglutination and phagocytosis, but also impact cell-to-cell communication and the related signal transduction.

## 5. Conclusions

To the best of our knowledge, the functional evaluations carried out in this study had not yet been performed on rainbow trout blood collected in K_3_EDTA and ACD-A. To fill this gap, we investigated and compared the impact of three different anticoagulants on the functions of rainbow trout PBLs. We also proposed a combined approach based on the simultaneous flow cytometric detection of viability, phagocytosis, and its related ROS production, allowing us to evaluate the anticoagulant that minimally alters the functional characteristics of the blood sample. Our results showed that Li-Heparin could be validly used in flow cytometric and microscopic approaches for all the morpho-functional assays we have investigated. In conclusion, in this study, we showed that the combined use of both flow cytometry and fluorescent microscopy can provide deeper insight into the influence of anticoagulants on morpho-functional characteristics of rainbow trout leukocytes. Nonetheless, the data obtained from this pilot study should be validated in larger-scale studies, with increased sample size.

## Figures and Tables

**Table 1:** Hematological count of rainbow trout whole blood collected in ACD-A, K_3_EDTA, and Li-Heparin.

	ACD-A	K_3_EDTA	Li-Heparin	*p*-Value
	Mean ± SD			
RBC (×10^6^/mm^3^)	1.07 ± 0.04 ^b^	1.13 ± 0.06 ^b^	1.64 ± 0.06 ^a^	0.0003
TBC (×10^4^/mm^3^)	3.78 ± 0.33 ^b^	4.48 ± 0.30 ^a^	4.51 ± 0.34 ^a^	0.008
WBC (×10^4^/mm^3^)	6.08 ± 0.14 ^a^	6.15 ± 0.19 ^a^	6.21 ± 0.15 ^a^	0.402
LC (×10^4^/mm^3^)	4.52 ± 0.2 ^a^	4.48 ± 0.34 ^a^	4.52 ± 0.16 ^a^	0.996
SL (×10^4^/mm^3^)	2.39 ± 0.17 ^a^	2.46 ± 0.13 ^a^	2.42 ± 0.09 ^a^	0.633
LL (×10^4^/mm^3^)	2.20 ± 0.12 ^a^	2.21 ± 0.10 ^a^	2.22 ± 0.11 ^a^	0.884
MON (×10^3^/mm^3^)	4.10 ± 0.07 ^a^	4.09 ± 0.13 ^a^	4.11 ± 0.09 ^a^	0.849
NEU (×10^4^/mm^3^)	1.12 ± 0.08 ^a^	1.11 ± 0.09 ^a^	1.07 ± 0.11 ^a^	0.738
EOS (×10^2^/mm^3^)	0.80 ± 0.01 ^a^	1.01 ± 0.01 ^a^	0.70 ± 0.01 ^a^	0.663

Notes: red blood cell (RBC), thrombocyte (TBC), white blood cell (WBC), total lymphocyte count (LC), small lymphocyte (SL), large lymphocyte (LL), monocyte (MON), neutrophil (NEU), and eosinophilic cell (EOS). Treatments with different superscript letters show significant differences across rows (Kruskal–Wallis’ test; *p* < 0.05; α = 0.017).

## Data Availability

The original contributions presented in the study are included in the article; further inquiries can be directed to the corresponding author.

## Abbreviations

The following abbreviations are used in this manuscript:

Li-Heparin	Lithium Heparin
K_3_EDTA	Tripotassium Ethylenediaminetetraacetic Acid
ACD-A	Acid Citrate Dextrose Formula A
ROS	Reactive Oxygen Species
FDA	Food and Drugs Administration
PBLs	Peripheral Blood Leukocytes
diH_2_O	Deionized Water
L15	Leibovitz’s L15
FCS	Fetal Calf Serum
FITC	Fluorescein Isothiocyanate
APC	Allophycocyanin
pHR	pHrodo
DAPI	4′,6-Diamidino-2-Phenylindole
RFU	Relative Fluorescence Unit
IQR	Interquartile Range
FSC	Forward Scatter
SSC	Side Scatter
MFI	Mean Fluorescence Intensity

## References

[1] Seibel H., Baßmann B., Rebl A. (2021). Blood Will Tell: What Hematological Analyses Can Reveal About Fish Welfare. Front. Vet. Sci..

[2] Fazio F. (2019). Fish Hematology Analysis as an Important Tool of Aquaculture: A Review. Aquaculture.

[3] Witeska M., Kondera E., Ługowska K., Bojarski B. (2022). Hematological Methods in Fish—Not Only for Beginners. Aquaculture.

[4] Nabi N., Ahmed I., Wani G.B. (2022). Hematological and Serum Biochemical Reference Intervals of Rainbow Trout, *Oncorhynchus mykiss* Cultured in Himalayan Aquaculture: Morphology, Morphometrics and Quantification of Peripheral Blood Cells. Saudi J. Biol. Sci..

[5] Saha N.R., Usami T., Saito T., Suetake H., Suzuki Y. (2002). Relationships between Immune and Reproductive Endocrine Systems in Fish. Fish. Sci..

[6] Řehulka J., Adamec V. (2004). Red Blood Cell Indices for Rainbow Trout (*Oncorhynchus mykiss* Walbaum) Reared in Cage and Raceway Culture. Acta Vet. Brno.

[7] Ates B., Orun I., Talas Z.S., Durmaz G., Yilmaz I. (2008). Effects of Sodium Selenite on Some Biochemical and Hematological Parameters of Rainbow Trout (*Oncorhynchus mykiss* Walbaum, 1792) Exposed to Pb^2+^ and Cu^2+^. Fish Physiol. Biochem..

[8] Kiron V. (2012). Fish Immune System and Its Nutritional Modulation for Preventive Health Care. Anim. Feed Sci. Technol..

[9] Segner H., Rehberger K., Bailey C., Bo J. (2022). Assessing Fish Immunotoxicity by Means of In Vitro Assays: Are We There Yet?. Front. Immunol..

[10] Neaga A., Lefor J., Lich K.E., Liparoto S.F., Xiao Y.Q. (2013). Development and Validation of a Flow Cytometric Method to Evaluate Phagocytosis of pHrodo^TM^ Bioparticles^®^ by Granulocytes in Multiple Species. J. Immunol. Methods.

[11] Parv K., Westerlund N., Merchant K., Komijani M., Lindsay R.S., Christoffersson G. (2021). Phagocytosis and Efferocytosis by Resident Macrophages in the Mouse Pancreas. Front. Endocrinol..

[12] Scatà M.C., Alhussien M.N., Grandoni F., Reale A., Zampieri M., Hussen J., De Matteis G. (2023). Hyperthermia-Induced Changes in Leukocyte Survival and Phagocytosis: A Comparative Study in Bovine and Buffalo Leukocytes. Front. Vet. Sci..

[13] Clark T.D., Donaldson M.R., Drenner S.M., Hinch S.G., Patterson D.A., Hills J., Ives V., Carter J.J., Cooke S.J., Farrell A.P. (2011). The Efficacy of Field Techniques for Obtaining and Storing Blood Samples from Fishes. J. Fish Biol..

[14] Cossarizza A., Chang H.D., Radbruch A., Abrignani S., Addo R., Akdis M., Andrä I., Andreata F., Annunziato F., Arranz E. (2021). Guidelines for the use of flow cytometry and cell sorting in immunological studies (third edition). Eur. J. Immunol..

[15] Lulijwa R., Alfaro A.C., Merien F., Meyer J., Young T. (2019). Advances in Salmonid Fish Immunology: A Review of Methods and Techniques for Lymphoid Tissue and Peripheral Blood Leucocyte Isolation and Application. Fish Shellfish Immunol..

[16] Faggio C., Arfuso F., Piccione G., Zumbo A., Fazio F. (2014). Effect of Three Different Anticoagulants and Storage Time on Haematological Parameters of *Mugil cephalus* (Linneaus, 1758). Turk. J. Fish. Aquat. Sci..

[17] Sheikh Z.A., Ahmed I. (2020). Comparative Evaluation of Two Anticoagulants Used for the Analysis of Haematological, Biochemical Parameters and Blood Cell Morphology of Himalayan Snow Trout, *Schizopyge plagiostomus*. Tissue Cell.

[18] Ciepliński M., Kasprzak M., Grandtke M., Steliga A., Kamiński P., Jerzak L. (2019). The Effect of Dipotassium EDTA and Lithium Heparin on Hematologic Values of Farmed Brown Trout *Salmo trutta* (L.) *Spawners*. Aquac. Int..

[19] Maqbool A., Ahmed I., Sheikh Z.A. (2014). Effects of Two Commonly Used Anticoagulants on Haematology and Erythrocyte Morphology of Rainbow Trout (*Oncorhynchus mykiss*). Int. J. Fish. Aquat. Stud..

[20] Witeska M., Wargocka W. (2011). Disodium EDTA Used as Anticoagulant Causes Hemolysis in Common Carp Blood. Turk. J. Vet. Anim. Sci..

[21] Gilor S., Gilor C. (2011). Common Laboratory Artifacts Caused by Inappropriate Sample Collection and Transport: How to Get the Most out of a Sample. Top. Companion Anim. Med..

[22] Mainwaring G., Rowley A.F. (1985). The Effect of Anticoagulants on *Blennius pholis* L. Leucocytes. Comp. Biochem. Physiol.—Part A Physiol..

[23] Walencik J., Witeska M. (2007). The Effects of Anticoagulants on Hematological Indices and Blood Cell Morphology of Common Carp (*Cyprinus carpio* L.). Comp. Biochem. Physiol.—C Toxicol. Pharmacol..

[24] Korcock D.E., Houston A.H., Gray J.D. (1988). Effects of Sampling Conditions on Selected Blood Variables of Rainbow Trout, *Salmo gairdneri* Richardson. J. Fish Biol..

[25] Smit G.L., Hattingh J., Schoonbee H.J. (1977). Observations on Some Effects of Disodium Ethylenediamine Tetra-Acetate and Heparin on Fish Blood. Comp. Biochem. Physiol. Part C Comp..

[26] Sussman L.N., Camacho D., Rosen E. (1971). Use of Adenine-ACD Solution in Long Term Storage of Blood. Am. J. Clin. Pathol..

[27] Betsou F., Gaignaux A., Ammerlaan W., Norris P.J., Stone M. (2019). Biospecimen Science of Blood for Peripheral Blood Mononuclear Cell (PBMC) Functional Applications. Curr. Pathobiol. Rep..

[28] Vallejos-Vidal E., Santillán-Araneda M.J., Goldstein M., Solarte-Murillo L.V., Maisey K., Reyes-Cerpa S., Vidal M., Reyes-Lopez F.E. (2025). Comparison of anticoagulant vacutainer blood collection tubes on rainbow trout (*Oncorhynchus mykiss*) leukocyte viability during long-term storage. Fish Shellfish Immunol..

[29] Hu Y., Maisey K., Subramani P.A., Liu F., Flores-Kossack C., Imarai M., Secombes C.J., Wang T. (2018). Characterisation of Rainbow Trout Peripheral Blood Leucocytes Prepared by Hypotonic Lysis of Erythrocytes, and Analysis of Their Phagocytic Activity, Proliferation and Response to PAMPs and Proinflammatory Cytokines. Dev. Comp. Immunol..

[30] Aizawa H., Kawabata H., Sato A., Masuki H., Watanabe T., Tsujino T., Isobe K., Nakamura M., Nakata K., Kawase T. (2020). A Comparative Study of the Effects of Anticoagulants on Pure Platelet-Rich Plasma Quality and Potency. Biomedicines.

[31] Kalb M.L., Potura L., Scharbert G., Kozek-Langenecker S.A. (2009). The Effect of Ex Vivo Anticoagulants on Whole Blood Platelet Aggregation. Platelets.

[32] Sachs L., Wesche J., Lenkeit L., Greinacher A., Bender M., Otto O., Palankar R. (2022). Ex Vivo Anticoagulants Affect Human Blood Platelet Biomechanics with Implications for High-Throughput Functional Mechanophenotyping. Commun. Biol..

[33] Carter P.H., Resto-Ruiz S., Washington G.C., Ethridge S., Palini A., Vogt R., Waxdal M., Fleisher T., Noguchi P.D., Marti G.E. (1992). Flow Cytometric Analysis of Whole Blood Lysis, Three Anticoagulants, and Five Cell Preparations. Cytometry.

[34] Minarova H., Palikova M., Mares J., Syrova E., Blahova J., Faldyna M., Ondrackova P. (2019). Optimisation of the Lymphocyte Proliferation Assay in Rainbow Trout (*Oncorhynchus mykiss*). Vet. Med..

[35] Ramsdorf W.A., de Guimarães F.S.F., Ferraro M.V.M., Gabardo J., da Trindade E.S., Cestari M.M. (2009). Establishment of Experimental Conditions for Preserving Samples of Fish Blood for Analysis with Both Comet Assay and Flow Cytometry. Mutat. Res.—Genet. Toxicol. Environ. Mutagen..

[36] Farías J.G., Herrera E.A., Carrasco-Pozo C., Sotomayor-Zárate R., Cruz G., Morales P., Castillo R.L. (2016). Pharmacological Models and Approaches for Pathophysiological Conditions Associated with Hypoxia and Oxidative Stress. Pharmacol. Ther..

[37] Wan X.S., Zhou Z., Ware J.H., Kennedy A.R. (2005). Standardization of a Fluorometric Assay for Measuring Oxidative Stress in Irradiated Cells. Radiat. Res..

[38] Wang J., Lei P., Gamil A.A.A., Lagos L., Yue Y., Schirmer K., Mydland L.T., Øverland M., Krogdahl Å., Kortner T.M. (2019). Rainbow Trout (*Oncorhynchus mykiss*) Intestinal Epithelial Cells as a Model for Studying Gut Immune Function and Effects of Functional Feed Ingredients. Front. Immunol..

[39] Park Y., Abihssira-García I.S., Thalmann S., Wiegertjes G.F., Barreda D.R., Olsvik P.A., Kiron V. (2020). Imaging Flow Cytometry Protocols for Examining Phagocytosis of Microplastics and Bioparticles by Immune Cells of Aquatic Animals. Front. Immunol..

[40] Al-Amoudi S.M. (2008). Anticoagulant Induced Leukoagglutination. Saudi Med. J..

[41] Deol I., Hernandez A.M., Pierre R.V. (1995). Ethylenediamine Tetraacetic Acid-Associated Leukoagglutination. Am. J. Clin. Pathol..

[42] Daves M. (2020). Hematology, Transfusion and Cell Therapy Unusual Leukoagglutination: A Rare Haematological Finding. Hematol. Transfus. Cell Ther..

[43] Haranath P. (2012). Patient Communication in Intensive Care Unit. Indian J. Crit. Care Med..

[44] Minarova H., Ondrackova P., Palikova M., Mares J., Blahova J., Jarova K., Faldyna M. (2021). Optimisation of Phagocytosis Assay in Rainbow Trout (*Oncorhynchus mykiss*). Vet. Med..

